# Love My Body: Pilot Study to Understand Reproductive Health Vulnerabilities in Adolescent Girls

**DOI:** 10.2196/16336

**Published:** 2020-03-30

**Authors:** Golfo Tzilos Wernette, Kristina Countryman, Kristie Khatibi, Erin Riley, Rob Stephenson

**Affiliations:** 1 Department of Family Medicine University of Michigan Ann Arbor, MI United States; 2 Center for Sexuality and Health Disparities, School of Nursing, University of Michigan Ann Arbor, MI United States; 3 Department of Systems, Population and Leadership School of Nursing University of Michigan Ann Arbor, MI United States

**Keywords:** adolescent, alcohol, sexually transmitted infections, risky sex, pregnancy

## Abstract

**Background:**

Sexually transmitted infections (STIs) are on the rise in the United States, and adolescent girls (15-19 years old) are more susceptible to acquiring STIs than their male peers. The co-occurrence of alcohol use and sexual risk taking contribute significantly to STI acquisition. Mobile health (mHealth) interventions are ideally suited for our target population and have demonstrated increases in STI testing in young people, as well as reductions in alcohol use.

**Objective:**

This pilot study used both qualitative and quantitative methods to explore the views of adolescent girls (age range 15-19 years old; 74.6%, 279/374 white) on the desired qualities and content of an mHealth app for sexual health.

**Methods:**

We conducted nine 60-min in-depth interviews (IDIs) to gather information and identify themes of sexual health and alcohol use, and we tested the feasibility of using a two-week social media campaign to collect survey information regarding sexual health risk in adolescent girls.

**Results:**

We iteratively coded IDIs and identified major themes around pressure of alcohol use, lack of STI knowledge, male pressure to not use condoms, and pregnancy as a worse outcome than STIs. Results from the web-based survey on risky health behaviors, which was completed by 367 participants, support the use of a sexual health app designed for girls.

**Conclusions:**

Future work will integrate these themes to inform the development of a culturally sensitive mHealth app to prevent STIs among adolescent girls.

## Introduction

### Background

Young people aged 15 to 24 years comprise half of all new sexually transmitted infections (STIs) in the United States, despite the fact that this age group accounts for just 27% of the sexually active population [1]. The Centers for Disease Control and Prevention recently reported that 25% of sexually active adolescent girls currently have an STI [1]. They also found that young women aged 15 to 24 years had the highest reported cases of chlamydia and gonorrhea, and that there was a 7.8% increase from 2016 in reported cases of primary and secondary syphilis. STIs are associated with significant morbidity and mortality, including pelvic inflammatory disease and infertility in women [2]. Adolescent girls are particularly vulnerable to STI acquisition compared with their male peers, as they face unique biological, social, and cultural vulnerabilities, such as increased cervical ectopy [1,3] and decreased power over sexual relationships [4,5]. There is a considerable need for a new paradigm in adolescent STI prevention efforts, particularly for girls, given the current data that suggest that prevention efforts do not seem to be effective.

### Alcohol Use and Sexual Health

The co-occurrence of alcohol use and sexual risk taking contribute significantly to STI acquisition, particularly in vulnerable populations, such as adolescent girls [6]. Alcohol continues to be the most widely abused substance by adolescents in the United States [7]. National survey data show that 42% of 10th graders and 62% of 12th graders reported lifetime use of alcohol [7], and 11% of females aged 12 to 17 years reported alcohol use in the last 30 days compared with 8.8% of males [8]. Adolescent girls who use alcohol and other substances are disproportionately more likely to engage in risky sexual behaviors that can result in STIs [6]. Owing to the interrelatedness of alcohol use and STI acquisition, and because both are associated with poor health outcomes, addressing alcohol use as a factor in sexual risk behavior may be particularly helpful for the development of sexual health interventions.

A number of evidence-based, sexual health programs delivered in school settings have demonstrated effectiveness in reducing risks during adolescence, including delaying sexual activity and increasing the consistent use of contraceptives [9]; however, there are structural and financial obstacles to the dissemination of these programs, and to maintain behavior changes. Furthermore, national surveys indicate that 83% of sexually experienced girls aged 15 to 17 years did not receive any formal sex education in school until *after* their first sexual experience [10].

### Sexually Transmitted Infections Knowledge and Testing

Sexual health knowledge is lacking among adolescents. One study found that 65% of 14 to 21-year-olds were aware of hepatitis, 57% were aware of human papillomavirus, and only 6.5% were aware of trichomoniasis [11]. In a recent study, adolescent girls who had been diagnosed with an STI were more knowledgeable about that particular STI compared with girls who had not been; however, their knowledge of other STIs were similar to girls who had never been diagnosed with one, suggesting that many adolescents do not learn about STIs until after they are diagnosed with one [12]. Limited STI knowledge among adolescents is associated with inconsistent condom use [13,14], multiple sex partners, delaying disease treatment, and failing to return for STI testing results [12]. There is a lack of STI testing among adolescents and young adults aged 15 to 25 years, with 42% of sexually active adolescents reporting not testing because they said they were “not at risk for an STIs”—an alarming statistic for a population that accounts for half of all new STI diagnoses [1,15].

### Mobile Health Interventions

Mobile health (mHealth) interventions use mobile phones or other wireless technology to disseminate health information in an effort to alter health behaviors and outcomes [16]. Owing to widespread smartphone and internet use, mHealth interventions are ideally suited for adolescents. A recent (2018) national survey noted that 9 out 10 teens go online multiple times daily, with 45% of teens saying they are online *almost constantly* [17]. Smartphone access was also high, with 95% of teens reporting they had access to a smartphone [17]. Content for an mHealth app can be standardized, tailored, and presented through interactive features, giving it the potential to have a wide reach, potentially leading to a greater impact at a lower cost [18]. Overall, mHealth interventions have demonstrated increased sexual health knowledge, increased STI testing and communication with clinicians about STI risk behavior, higher rates of condom and contraception use, lower rates of unprotected sex, and reductions in alcohol use among adolescents [16,19-21].

Despite their promise, there is still a dearth of knowledge about efficacy, communication best practices for mHealth interventions, and a need for further research into effective content creation and health promotion messaging [16,22]. Furthermore, there is a lack of evidence-based preventive interventions that specifically target both sexual health and alcohol use among adolescent girls aged 15 to 19 years, a particularly vulnerable group [23,24]. This pilot study sought to fill these gaps by conducting in-depth individual interviews and anonymous surveys with female adolescents to (1) understand attitudes about sexual health and alcohol use, and (2) to determine preferences for content, messaging, and format for an mHealth app aimed at reducing risky sexual health behaviors and alcohol use.

## Methods

### Design

This pilot study used both qualitative and quantitative methods to explore the views of adolescent girls on the desired qualities and content for an mHealth app on sexual health and alcohol use. The first phase of the study was qualitative, in which we conducted in-depth interviews (IDIs), and the second phase was quantitative, in which we conducted web-based surveys using a social media–recruited sample. We recruited adolescent girls aged 15 to 19 years who met study inclusion criteria to participate in a 1-hour focus group. Inclusion criteria included endorsing sexual risk behavior (eg, unprotected sex) and alcohol use within the last 30 days. Owing to recruitment challenges, including a lack of study screener completion, lack of girls reporting both risk factors, and difficulty scheduling participants for focus groups, we changed the study format to individual IDIs and modified our inclusion criteria to include adolescent girls aged 15 to 19 years who endorsed *either* a sexual behavior risk (eg, unprotected sex) or an alcohol behavior risk in the last 30 days. This study was approved by the Institutional Review Board of the University of Michigan.

### Recruitment

Recruitment for the focus groups took place between August 2017 and December 2017, and IDI recruitment took place between January 2018 and April 2018. Recruitment for the web-based survey occurred in July 2018. For the qualitative phase of this study, we used both active and passive recruitment methods, including onsite recruitment at an urban family medicine clinic that is the primary care provider for an underserved population of children, adolescents, and adults. Approximately 55% of the clinic’s patients identify as Latina or African American. Participants were recruited in the waiting room before their scheduled appointment. Participants were approached, the study was described, and if they expressed interest, they were asked to take the screener. Interested participants provided verbal assent for the screener and received a small gift (eg, lip balm) for participating. Eligible girls were invited to participate in an individual IDI. Participants received US $30 cash for completing the IDI.

We included passive recruitment to the study design to enhance recruitment efforts. Passive recruitment included advertising on a large public university campus in the Midwest, at local clinics, and on college listservs in a broader population area compared with active recruitment. Interested participants reached out via email and received a link to the screener; those who met inclusion criteria were invited to participate in an IDI.

In the second phase of the study, we tested the feasibility of using social media (eg, Facebook) to recruit a national sample of at-risk adolescent girls for research studies, collect survey data on sexual health risk and substance use, and gauge interest in a potential mobile phone app that addresses these issues. We ran social media ads on Facebook and gathered survey data for a period of 2 weeks. We targeted the ad by age (15-19 years old), gender (female), location (the United States), demographics (eg, education level, life events, and relationship status), interests (eg, entertainment and pop culture), and behaviors (eg, shopping). The survey was conducted through Qualtrics software (Qualtrics, Provo, UT) with no confidential information such as names, addresses, or Internet Protocol addresses collected. Participants who completed the social media survey did not receive an incentive.

### Instruments

#### Screener

Participants completed a brief (10-min) health screener via an iPad to determine study eligibility. The screener consisted of questions measuring substance use risk behaviors from the CRAFFT Screening Tool for Adolescent Substance Abuse (computer version), a well-validated and reliable measure for this population recommended by the American Academy of Pediatrics’ Committee on Substance Abuse for use with adolescents [25]. It included questions about the use of alcohol, marijuana and other illicit drugs, over the counter drugs, and prescription drugs. Items assessing sexual risk behavior asked about recent vaginal and anal intercourse, including intercourse with and without condoms in the past 30 days.

#### In-Depth Interviews

Two members of our research team with previous experience with qualitative and adolescent research conducted nine 60-min IDIs. The semistructured interview guide consisted of open-ended questions focused on drinking habits, sexual health behaviors, effective messaging, social media and app preferences, as well as STI knowledge and level of STI concern. Questions such as “How much alcohol do you or other girls your age drink?” and “How are drinking and unprotected sex related for girls your age?” were used to assess typical adolescent female behavior, while “What do you think you or other girls your age want to know about preventing STIs?” and “What features would you like to see in a health app?” helped to determine content and preferences for an mHealth app. Interviews were audio recorded and transcribed verbatim using the web-based transcription service, Scribie. The interview transcripts were reviewed by research staff to ensure accuracy.

#### Demographic Survey

A post-IDI demographic survey was emailed to the 9 participants to determine age, income, sexual orientation, ethnicity, sex, and education level. To ensure confidentiality, this demographic survey data were not linked to the participant’s IDI data.

#### Qualitative Data Analysis

ATLAS.ti (Scientific Software Development GmbH, 2013-2018) was used to code the interview transcripts. A codebook was iteratively created and agreed upon by all research team members after assessing the major themes from the interview transcripts. The codebook consisted of seven major alcohol and sexual health–related themes such as, *Drinking behaviors* and *STIs & pregnancy*. Within these major themes, we had six subcodes for more specificity such as, *Binge drinking* and *Condom*. Two researchers (KC and KK) independently coded all transcripts and differences were reconciled in-person to create a final coded dataset for the qualitative analysis. We determined that saturation had been achieved after nine IDIs: analysis demonstrated that we were no longer identifying new themes.

#### Web-Based Survey

We conducted an anonymous, web-based survey using Facebook to test the feasibility of this recruitment method. Survey questions included demographics, substance use questions (eg, “Do you ever use alcohol or drugs to relax, feel better about yourself, or fit in?”), and sexual health questions (eg, “In the past 30 days, how often did you use condoms when you had sex?”). The survey also included a question on whether or not respondents would use a sexual health app designed for girls.

## Results

### In-Depth Interview Participants

Background characteristics of the IDI participants are shown in Table 1. Recruitment occurred in two separate phases. During the first recruitment phase, we approached 61 girls through active (clinic-based) recruitment, screening a total of 50 girls over the course of 5 months. Of the 50 girls screened, only 7 (14%) were eligible, but no eligible girls participated in a focus group discussion.

Phase 2 of recruitment was conducted both via active (clinic-based) and passive recruitment strategies. A total of 15 girls were approached during active recruitment, of which 7 completed the screener, and 4 (57%) were eligible. Through our passive recruitment strategies, 19 girls contacted us, 18 completed the screener, and 50% (9/18) were eligible. All 9 of these participants completed an IDI. A CRAFFT score was determined using the screener data for each participant. The range of scores was from 0 to 4, with a score of 4 or more indicating possible substance dependence.

**Table 1:** Study characteristics of in-depth interview participants (N=9).

Variables		Value
Age (years), mean (SD), range		18.5 (0.52), 18-19
**Race/ethnicity^a^ (n=7), n (%)**		
	White	4 (57)
	Biracial	2 (28)
	Other	1 (14)
	Hispanic or Latina/Latinx	2 (28)
**Highest level of education^a^ (n=7), n (%)**		
	Currently in college or another training program	7 (100)
**Family household income^a^ (US $; n=7), n (%)**		
	35,000-49,999	1 (14)
	50,000-74,999	1 (14)
	200,000 or more	3 (42)
	Not sure	2 (28)
**Sexual orientation^a^ (n=7), n (%)**		
	Straight/heterosexual	6 (85)
	Gay/lesbian/homosexual	1 (14)
**Current relationship status (n=9), n (%)**		
	Single	4 (44)
	In a relationship	5 (55)
**Condom use in the last 30 days (n=9), n (%)**		
	I have not had sex in the last 30 days	1 (11)
	All of the time	2 (22)
	Most of the time	4 (44)
	Some of the time	1 (11)
	None of the time	1 (11)
**Any alcohol use in the last 12 months (n=9), n (%)**		
	Yes	9 (100)
**Any marijuana use in the last 12 months (n=9), n (%)**		
	Yes	4 (44)
**CRAFFT^b^ score^c^ (n=8), n (%)**		
	0	2 (25)
	1	1 (12)
	2	2 (25)
	3	1 (12)
	4	2 (25)

^a^Only 7 of the 9 participants completed demographic questions.

^b^CRAFFT: CRAFFT Screening Tool for Adolescent Substance Abuse (computer version).

^c^Only 8 of the 9 participants completed the CRAFFT assessment.

### Web-Based Survey Participants

Participant characteristic results from the web-based survey are presented in Table 2. Respondents were asked questions on their relationship status and condom use, as well as their alcohol and drug use (see Table 3). The majority of respondents (86%; 314/367) reported that they would use a sexual health phone app for adolescent girls. Surveys were considered complete and included in the analysis if all questions were viewed by the participants. Participants could choose not to respond to questions that made them feel uncomfortable, and their surveys were still included in the analysis.

**Table 2:** Study characteristics of web-based survey participants (N=367).

Variables		Value
Age (years), mean (SD), range		16.2 (0.88), 15-19
**Race (n=365), n (%)**		
	White	275 (75.3)
	Biracial	26 (7.1)
	African American/black	25 (6.9)
	Asian	20 (5.5)
	Native Hawaiian or Pacific Islander	5 (1.4)
	Native American or Native Alaskan	3 (0.8)
	Other^a^	11 (3.0)
**Ethnicity (n=367), n (%)**		
	Hispanic or Latina/Latinx	33 (9.0)
**Highest level of education (n=367), n (%)**		
	Currently in high school	335 (91.3)
	Graduated high school of have GED^b^	15 (4.1)
	Not in high school and did not complete high school or GED	2 (0.5)
	Currently in college or another training program	15 (4.1)
**Family household income (n=361) (US $), n (%)**		
	Less than 25,000	24 (6.7)
	25,000-34,999	21 (5.8)
	35,000-49,999	23 (6.4)
	50,000-74,999	36 (10.0)
	75,000-99,999	32 (8.9)
	100,000-149,999	38 (10.5)
	150,000-199,999	14 (3.9)
	200,000 or more	14 (3.9)
	Not sure	159 (44.0)
**Sexual orientation (n=365), n (%)**		
	Straight/heterosexual	123 (33.7)
	Bisexual	153 (41.9)
	Gay/lesbian/homosexual	39 (10.7)
	Uncertain or questioning	50 (13.7)
**Current relationship status (n=366), n (%)**		
	Single	221 (60.4)
	In a relationship, but not married	145 (39.6)

^a^Other: write-in responses for race included the following: Hispanic (2), North African (1), Middle Eastern (2), Multiracial (1), Native American/African American/Eastern European (1), Other or no specification (3).

^b^GED: General Education Development.

**Table 3:** Sex risk and substance use behavior responses of web-based survey participants (N=367).

Variables		Value, n (%)
**Condom use in the last 30 days (n=362)**		
	I have not had sex in the last 30 days	274 (75.7)
	All of the time	13 (3.6)
	Most of the time	12 (3.3)
	Some of the time	13 (3.6)
	None of the time	50 (13.8)
**Condom use in the last 30 days among sexually active participants (n=88)**		
	All of the time	13 (15)
	Most of the time	12 (14)
	Some of the time	13 (15)
	None of the time	50 (57)
**Any alcohol use in the last 12 months (n=367)**		
	Yes	123 (33.5)
**Any marijuana use in the last 12 months (n=367)**		
	Yes	83 (22.6)
**Any other drug use in the last 12 months (n=366)**		
	Yes	32 (8.7)
**Ever ridden in a car driven by someone (including yourself) who was *high* or had been using alcohol or drugs? (n=367)**		
	Yes	89 (24.2)
**Ever use alcohol or drugs to relax, feel better about yourself, or fit in? (n=365)**		
	Yes	86 (23.6)
**Ever use alcohol or drugs while you are by yourself or alone? (n=367)**		
	Yes	80 (21.8)
**Ever forget things you did while using alcohol or drugs (ie, *blacking out*)? (n=366)**		
	Yes	35 (9.6)
**Do family or friends ever tell you that you should cut down on your drinking or drug use? (n=366)**		
	Yes	12 (3.3)
**Have you ever gotten in trouble while using alcohol or drugs? (n=367)**		
	Yes	17 (4.6)
**Would you use a sexual health app for girls? (n=367)**		
	Yes	314 (85.8)

### In-Depth Interview Results

Data from the nine IDIs resulted in four major interrelated themes described in the sections below. These themes included alcohol and college life, alcohol and condomless sex, STIs and pregnancy, and technology use and app preferences.

#### Alcohol and College Life

Participants agreed that alcohol plays a big role in college life, whether or not one chooses to drink. They described both alcohol and the pressure to drink as omnipresent. Several interrelated reasons were given for drinking, and these reasons were often linked with a desire to relieve school and/or social stress and anxiety. However, the most cited reason for drinking was the pressure to drink received from peers, sexual partners (potential and actual), and from society/media as a whole.

Most of the drinking behaviors the participants described would be classified as binge drinking (defined as drinking more than 4 drinks in 2 hours for women, and more than 5 in 2 hours for men), and/or heavy drinking (defined as binge drinking on 5 or more days within a month, the National Institute on Alcohol Abuse and Alcoholism [26]). This kind of drinking was described as the norm. Some even said they felt that binge drinking was a *serious problem* on campus. When *partying* was described, they spoke mostly of alcohol use. Drug use other than alcohol was rarely mentioned in the interviews, even though one participant described drugs as being *prominent* on campus, and that *all sorts* of drugs were mixed with alcohol at parties.

#### Alcohol and Condomless Sex

When asked about the relationship between alcohol and condomless sex, most said that these were *strongly connected* because drinking too much lowers inhibition and impairs decision making, often leading to *bad* decisions. But the participants also provided a more nuanced look into the correlation of alcohol use and condomless sex. Specifically, the reasons why it happens in the first place, and how alcohol use intensifies it. These reasons overlapped and tended to reinforce each other, including male pressure for condomless sex, lack of female sexual empowerment, the societal stigma of female sexuality, and a lack of concern or knowledge of STIs.

The persistent reason for condomless sex was said to be the pressure that young women receive from young men to not wear one. Five of the nine participants explained how drinking alcohol made it easier for young men to convince young women to not use condoms:

... 'Cause when you're intoxicated, you, uh, aren't gonna be thinking completely straight and you might just be relying on emotional decisions at that point…And I think with, you know, like rape culture and kinda like the way sex is depicted in society today for young guys in college. I think that women, uh, are pressured to have unprotected sex sometimes...

Interview 3, Age 19

Ignorance surrounding the prevalence of STIs was another reason given for condomless sex. Participants repeatedly said that their peers did not talk about STIs, and they believed they were not really a problem, because STIs were not that prevalent among young people. Similar to condom use, issues of trust came up when talking to a partner about STIs. The consensus was you do not talk to your sexual partner about STIs or their status; to do so would be an implication of mistrust, and a belief that they are not *clean*.

I've always had, like made sure that the... Like, my current partner does not have any STDs or STIs. But I know that, umm, a lot of my friends have been like, ‘Oh my God! You asked him that? How could you do that? Like, isn't that you don't trust him?’ I'm like, ‘No, it's that I want my body to be safe. I'm clean, so I wanna make sure he's clean.’

Interview 9, Age 18

#### Sexually Transmitted Infections and Pregnancy

Two resounding themes came out of our questions on STIs and pregnancy: (1) young women are largely ignorant of STIs and their health effects, and (2) young women are not that concerned about STIs, but rather, about pregnancy. When asked what they knew about STIs, the majority of our participants answered that they had limited knowledge. A few participants knew the names of some STIs and knew that they were transmitted sexually, but unconfidently followed-up their responses with an admission of *not really knowing much* about them. Participants said they would like to know more about the etiology of the different STIs, how to effectively prevent them, and the associated treatment, but also said that the main concern in their sex lives was to avoid pregnancy.

The lack of concern about STIs was often attributed to the belief that young women simply did not realize how common these infections were. Some expressed ignorance because they had never had an STI, and their friends had not either. This was coupled with beliefs that STIs were a “thing that older people have,” not something for women their age to worry about because, “he's what, like 19 you know, he surely hasn't gotten around that much you know, that he has an STD already.” None of the participants felt that the information they had received in high school was adequate, and some even said that what they had been told was completely inaccurate. Multiple participants reported having had abstinence-based sexual education class, and they felt that these classes were not very informative and relied heavily on scare tactics. One participant said her sex-ed teacher told the class that condoms “wouldn’t protect against STIs,” and were “only 50% effective at preventing pregnancy.”

Participants also said that young men did not believe that STIs were prevalent, and therefore did not think condoms were that important. They said that young men generally preferred the withdrawal method. As they believed that STIs were not prevalent in young people, participants admitted having feelings of invincibility, and trusted that they would either know if their sexual partner had an STI, or that their sexual partner would communicate if they had an STI:

I think it's more just the like, ‘it would never happen to me’ kinda idea. I mean like I'm guilty of it, like I definitely think, ‘Oh that would never happen to me’, like I wouldn't have sex with someone who wouldn't tell me if they have an STD, umm like I'm smarter than that, I'm not gonna do it. Umm but in reality, I'm sure it could happen to anyone.

Interview 3, Age 19

Participants agreed that getting pregnant would be a far *worse* outcome than getting an STI, because of the social stigma surrounding teenage/young mothers, and what they described as the *longer term consequences* of pregnancy. These *consequences* persisted whether the pregnancy was carried to term or not. Abortion was referred to as *scary*, *very painful,* and *serious procedure*, and an emotionally difficult choice to make. Carrying the pregnancy to term was also described as an emotionally difficult choice to make; thus, avoiding pregnancy altogether was of the utmost importance. Participants acknowledged that there is a stigma surrounding STIs and condomless sex but said that pregnancy had more visible (and therefore, public) consequences, while STIs could more easily be treated and kept a private matter. They also saw STIs as a problem affecting both parties; something they would go through together. Pregnancy, on the other hand, was something they feared they would face alone. As pregnancy is something that only physically affects women’s bodies, participants said that men did not see it at as something that affects them much; that it was ultimately a woman’s problem:

...I've had girlfriends that have gotten pregnant and it's affected their lives deeply. But the guy, it affected their life, like zero.

Interview 9, Age 18

#### Technology Use and App Preferences

All participants reported smartphone use and were actively engaged with multiple mobile apps. The most frequently used apps were for email, music, videos (eg, YouTube), and social media. Although not every participant used the same social media apps, all participants reported using at least one social media app. When asked to reflect on specific app features they liked, they said an app needs to be interactive, visually attractive, easy to use, have tracking capabilities, and the ability to set goals and earn rewards.

When asked about sexual health–focused apps, none of the participants were familiar with any. Some participants mentioned using a period-tracking app, which they said sometimes included information on STIs and contraception. When asked about alcohol-related apps, several participants discussed a drinking app created by the university that was focused on reducing alcohol consumption to *stay safe* and *in control*.

#### Effective Messaging

To determine the most compelling way to present messaging around safer sex and reducing or avoiding alcohol/drugs in an mHealth app, we asked participants what effective and ineffective advertising or messaging they have seen. They reported having heard some effective messaging but stressed the need for more and different messages, especially surrounding sexual health. When asked to describe the kind of messaging they felt would work best with their peers, there was some disagreement about whether to use *scare tactics* in alcohol-related messaging, but the participants agreed that scare tactics needed to be avoided in sexual health messaging. They emphasized wanting to know facts and ways to stay safe when it came to sex and alcohol use, rather than just receiving messages of abstinence.

### Effective Alcohol Use Messaging

Participants overwhelmingly said that they avoided messages in the *don’t drink* genre. They saw them as ineffective and were generally dismissed because they are seen as being unrealistic and out-of-touch:

I feel like when they promote not drinking whatsoever, people will be like, ‘Yeah, right, whatever,’ because it's gonna happen, girls are going to drink. You're in college. Umm, there's no doubt about it.

Interview 6, Age 18

Including practical messaging to reduce alcohol consumption was thought to be the most effective approach, such as highlighting the benefits of drinking less (ie you can still have fun but avoid hangovers). Health and safety came up frequently when discussing alcohol-related topics that participants would like to see in an app. Specifically, they said they want to learn about *healthy levels* of drinking versus *problem drinking*, and how to gage and manage their own personal alcohol limits. They recommended that our app contain a drink-tracking feature like the university’s app discussed above. They also said it would be helpful to include a centralized list of resources that provide substance use help.

Participants resoundingly said that they wanted an app that focuses on helping young women, not on shaming them. To them, this meant that the app would use positive language, and would validate young women’s desire to try alcohol as something that was normal and not as something that was *bad* or *immoral*. Validating that it was equally normal to *not* want to drink was something they asked to include as well as emphasizing that if they did choose not to drink, they would not be alone in their choice.

### Effective Sexual Health Messaging

Similar to alcohol-related messaging, participants said that the most effective way to talk to young women about sexual health was to be factual in an empowering manner, not in a moralizing or judgmental one. One participant said that when issues of women’s sexual health were put in a moralizing manner, it was too “easy to cross into the slut shaming line,” which to her, made them ineffective. She further discussed how she disliked her high school sexual education class because it centered around scare tactics:

Definitely listing all the symptoms is fine, but the pictures I feel like can be overly graphic... But like the way it was taught to me, it was like, you have sex you get an STI, like a direct link there. So like basically you have sex, you're dirty, even if you don't get an STI.

Interview 9, Age 18

When we asked where young women went for information on STIs, most participants said that they generally did not search for information on STIs. They said that STIs were just not something people their age worried much about. Overall, participants desired detailed information on STIs, not just how to prevent them. They wanted to know how they can affect your body and overall health, which ones can be cured and which ones cannot, how the testing process works for different STIs, and what the implications are for their sexual partner(s) with a positive test.

Participants explained that an app should also include information on sexual consent, coercion, and issues of sexual assault and how to prevent it. As many women become sexually active during adolescence and into college, they thought it was very important to include the definition of consent and how to give it. They also wanted the app to have information on how to recognize and get out of potentially dangerous situations, and steps to mitigate and navigate a sexual assault situation. Finally, participants said they wanted empowering messages about their sexuality and their bodies. They said that young women want to be in control of their sex lives without experiencing stigmatizing labels. They want to be comfortable about their sexuality and comfortable using condoms. As one young woman put it:

I think deep down, all women would prefer to be protected.

Interview 3, Age 19

## Discussion

### Principal Findings

This pilot study used both qualitative and quantitative methods to explore the views of adolescent girls on the desired qualities and content for an mHealth app on sexual health and alcohol use. Overall, the results of this pilot work were encouraging. We identified and recruited adolescent girls who endorsed recent alcohol use and sexual risk behavior to participate in the individual IDIs. Our study was feasible with respect to the study procedures, and passive recruitment methods were most effective in identifying girls who endorsed these risks. Moreover, we found that using social media to recruit and engage adolescent girls who endorsed high-risk behaviors was feasible and acceptable. Of the 382 individuals who started the survey, 367 (96.0%) completed it. Moreover, 86% (314/367) endorsed that they would use a sexual health app for girls.

Several themes emerged from the IDIs, with the major themes involving alcohol and college life, alcohol and condomless sex, STIs and pregnancy, and technology use and preferences. Participants offered, from their personal experiences as well as those of others, their perspectives supporting that alcohol use and sexual risk behavior are closely connected in this phase of life. The co-occurrence of alcohol use and sexual risk taking contribute significantly to STI acquisition, particularly in vulnerable populations, such as adolescent girls.

There was a general sense of inexperience with STIs and, subsequently, a lack of knowledge on prevention and vulnerability. Two very clear themes emerged during our interviews with respect to our questions on STIs and pregnancy. First, young women are largely ignorant of STIs and their health effects, and second, young women are relatively unconcerned about STIs, but rather, extremely concerned about pregnancy. The deep-seated concern about pregnancy was not only because of the significant life change it would bring but also because of the moral implications in a society where having a teenage pregnancy brings harsh judgment and shame. STIs were seen as something that could easily be hidden if you contracted one, but pregnancy was something out in the open. In our IDIs, we found participants to be open about recommendations for a sexual health app, for example, “if you want to talk to young people about STIs, focus on *why* young people should prevent them, not just *how* they can prevent them.”

### Comparison With Previous Work

The use of mHealth interventions to target sexual health and substance use among youth holds promise in reaching large numbers of at-risk adolescents remotely and to tailor content to individual user needs. In 2017, a systematic review of mHealth interventions for alcohol/drug use prevention among young adults found mixed results among the individual studies, but an overall support for the efficacy of mHealth interventions in reducing substance use. They included a variety of mHealth modalities such as text messaging, web-based apps, and smartphone apps. The limitations of these studies were largely because of small sample sizes, lack of data on long-term effects, lack of examination into potential gender differences in intervention effects, and lack of diversity in participant age and environment [27].

Our review of federal registries for evidence-based interventions (EBIs) on adolescent substance use prevention and adolescent sexual health found none that focused on both of these health risks simultaneously for nontreatment seeking or nonincarcerated adolescent girls [23,24]. While there were several EBIs focused on adolescent girls’ sexual health [28-33], there was a dearth of EBIs for substance use prevention in the 15 to 19 age range, and none that focused on young women. Furthermore, the sexual health EBIs were either specific to clinic-seeking, at-risk, or racial and ethnic minority adolescent girls. The sexual health EBIs with the most promising results still faced the limitation of reach and scalability; none of them were mHealth, and all relied on a clinic, classroom, or community-based setting with trained facilitators [28-33]. Not only would a mobile sexual health app be able to reach more adolescents but it would also be more cost-effective and provide greater impact.

### Strengths and Limitations

This study included a number of strengths. We explored views on a topic of great public health significance and successfully engaged youth to share their attitudes and feelings on sensitive topics. We used social media as a tool to engage high-risk adolescents to collect informative data, demonstrating that this is a suitable platform for our future work. Owing to our study population comprising of college-enrolled female adolescents, there are still gaps in understanding the preferences and needs of female adolescents from disadvantaged backgrounds and the results cannot be generalized to the population at large. The inability of this study to successfully recruit girls to complete focus groups exemplifies the challenges that need to be understood among this population to best reach them effectively.

### Conclusions

This pilot study demonstrates a clear need to reach, inform, and empower adolescent girls on the topics of sexual health and alcohol/drug use. Given the escalating rates of STI acquisition that continues to affect this vulnerable group, addressing the co-occurrence of alcohol/drug use and sexual risk taking is a research area of high priority, and also for which data are insufficient and scarce. Future work will integrate these areas to inform the development of a culturally sensitive mHealth app to empower adolescent girls and to reduce health risks and prevent STIs among them.

## Abbreviations

EBI: evidence-based intervention
IDI: in-depth interview
mHealth: mobile health
STI: sexually transmitted infection
